# Frizzled 2 Functions in the Regulation of TOR-Mediated Embryonic Development and Fecundity in *Cyrtorhinus lividipennis* Reuter

**DOI:** 10.3389/fphys.2020.579233

**Published:** 2020-09-16

**Authors:** Linquan Ge, Lu Jiang, Sui Zheng, Yongkai Zhou, Qing Wu, Fang Liu

**Affiliations:** School of Horticulture and Plant Protection, Yangzhou University, Yangzhou, China

**Keywords:** *Cyrtorhinus lividipennis*, Frizzled 2, target of rapamycin pathway, Wnt/β-catenin pathway, fecundity

## Abstract

The mirid bug, *Cyrtorhinus lividipennis* Reuter, is an important predator of rice planthoppers in Asia. In a previous study, *C. lividipennis* fed on gramineous weeds with brown planthopper (BPH) eggs had reduced development compared to those fed on rice with BPH eggs. In the current study, the concentrations of selected amino acids (AAs) were higher in rice than five gramineous species, which might explain the enhanced growth of *C. lividipennis* on rice. When *C. lividipennis* was fed on AA-deprived artificial diets, the Wnt/β-catenin pathway was inhibited. Furthermore, *C. lividipennis* females silenced for expression of *Frizzled 2* (*Fz2*) showed a significant reduction in the Wnt/β-catenin and target of rapamycin (TOR) pathways. Silencing *Fz2* led to decreased expression of the vitellogenin gene (*Vg*), lower Vg accumulation in oocytes, reduced soluble protein in ovaries and fat bodies, reduced titers of juvenile hormone, prolonged preoviposition periods, and lower predation capacity, body weight, and egg numbers as controlled to controls. *Fz2* silencing resulted in undeveloped ovaries and the inhibition of oocyte growth in the ovarioles, resulting in decreased numbers of offspring and reduced hatching rates. The silencing of *Fz2* also resulted in aberrant embryos with undeveloped eyespots and organs, suggesting that *Fz2* is an essential gene for embryonic development, oogenesis, and egg maturation. In summary, this study established a potential link between Wnt and TOR pathways, which interact synergistically to regulate *C. lividipennis* reproduction in response to AA signals. These results provide valuable new information that can be applied to large-scale rearing of *C. lividipennis* predators, which is important for reducing planthopper damage in rice fields.

## Introduction

The mirid bug, *Cyrtorhinus lividipennis* Reuter (Hemiptera: Miridae), is prevalent in rice fields and is an important predator of leafhoppers and planthoppers, including the brown planthopper (BPH), *Nilaparvata lugens* Stål (Hemiptera: Delphacidae). *C. lividipennis* feeds on planthopper eggs and nymphs in irrigated rice fields (Katti et al., 2007; Sigsgaard, 2007). Predacious *C. lividipennis* nymphs can consume approximately 7.5 eggs or 1.4 nymphs per day over a 2-week period, and adults can consume 10 eggs, 4.7 nymphs, or 2.4 adults per day over a 10-day period (Reyes and Gabriel, 1975); thus, a single bug can consume up to 66 nymphs during its 24-day life span. Previously, *C. lividipennis* was shown to feed on BPH eggs inhabiting different gramineous species growing in the perimeter of rice fields; however, longevity, development, and reproduction were reduced on gramineous species when compared to rice plants (Yu et al., 1996). One possible explanation for this finding is that rice is more nutrient dense than the surrounding gramineous species, and the enriched nutritional content of rice might promote greater survival and development of *C. lividipennis* in comparison to gramineous weeds. One transducer of nutritional signals is the target of rapamycin (TOR) pathway, which transmits amino acid (AA) signals (Fingar and Blenis, 2004; Attardo et al., 2006; Loewith and Hall, 2011) and regulates vitellogenesis *via* the *Vg* gene (Hansen et al., 2004, 2005). When TOR was inhibited by rapamycin or RNAi, *Vg* expression and egg production were inhibited in the mosquito, *Aedes aegypti* (Hansen et al., 2005). The AA-dependent TOR signaling pathway was shown to mediate the phosphorylation of S6K kinase (Hansen et al., 2005), which is a critical regulator of *Vg* expression (Attardo et al., 2003; Park et al., 2006).

*Wnt1*, which was originally named *Int-1*, was first identified in mouse cancer cells (Nusse and Varmus, 1982). In a subsequent report, a homolog of *Wnt* named *Wingless* was identified in *Drosophila* (Rijsewijk et al., 1987) and was shown to function in fly development as part of a developmental cascade (Vinson et al., 1989; Clevers, 2006). The glycoprotein Wnt and Frizzled (Fz), a transmembrane receptor for Wnt, interact at the cell surface as part of a signal transduction pathway that regulates gene expression (Logan and Nusse, 2004; Sen and Ghosh, 2008). In canonical Wnt signaling pathways, Wnt ligands bind to Frizzled/low-density lipoprotein receptor-related protein (LRP), which activates Dvl (scaffold protein) and results in the inactivation of glycogen synthase kinase 3 (GSK3β) and stabilization of the cytosolic co-activator protein, β-catenin (Clevers, 2006). The accumulation of β-catenin in the cytosol leads to translocation into the nucleus, thus causing the formation of the transcriptionally activated TCF (T cell factor) complex at the promoter regions of target genes (Clevers, 2006). In contrast, non-canonical Wnt signaling depends on intermediary proteins, such as calmodulin kinase (CamK) and protein kinase C (PKC), which do not require β-catenin (Logan and Nusse, 2004; Gordon and Nusse, 2006; Mikels and Nusse, 2006; Sen and Ghosh, 2008; Mosimann et al., 2009). *Drosophila* Fz2 was previously shown to modulate embryonic patterning (Kennerdell and Carthew, 1998). In the mosquito *Anopheles gambiae*, which vectors the malarial parasite *Plasmodium berghei*, Fz2 triggered melanization in the mosquito midgut after infection with *P. berghei* (Shiao et al., 2006). More recently, Fz2 was shown to function with the TOR pathway in egg production by the mosquito *Aedes aegypti* (Weng and Shiao, 2015).

Although multiple intermediary proteins have been identified in the Wnt signaling pathway (Behura et al., 2011), the function of Fz2 in transducing AA signals in *C. lividipennis* is unknown. In this study, we investigate the involvement of Wnt/β-catenin and TOR pathways in response to AAs signals in *C. lividipennis* and their potential role in reproduction. Our results may eventually result in an optimized artificial diet for large-scale rearing of *C. lividipennis*, which would improve our ability to deploy the predator for BPH control in rice fields.

## Materials and Methods

### Measurement of Amino Acid Concentrations

Stems (0.1 g fresh weight) were removed from rice (40 days) and five gramineous (40 days) species (*Digitaria ciliaris*, *Cynodon dactylon*, *Leptochloa chinensis*, *Elusine indica*, and *Echinochloa glabrescens*) and transferred to 1 ml of 6 mol/L HCl for 15 min. Suspensions were allowed to settle for 15 min at room temperature, and an aliquot (0.5 ml) was centrifuged at 10,000 × g to produce a clear extract. AAs were extracted using the EZ:Fast Free Amino Acid kit (Phenomenex, CA, Unitd States). Gas chromatography (GC)–mass spectrometry (MS) analysis of the extracted samples was conducted as described (Curtis et al., 2016). AA standards were supplied with the EZ:Fast kit, and calibration curves were calculated for each AA. Data were analyzed with Agilent 5975 software.

### Insect Rearing

*C. lividipennis* was collected from rice fields located at Yangzhou University, Yangzhou, China. Mirid bug colonies were reared in a controlled environmental chamber maintained at 26 ± 2°C and 75% relative humidity (RH) with a 16:8 h light:dark photoperiod for multiple generations. *C. lividipennis* were supplied with BPH eggs as a food source.

### Quantitative Real-Time PCR

Total RNA was isolated from *C. lividipennis* with Trizol reagent (Invitrogen, Carlsbad, CA, United States) and treated with RNase-free DNase I. cDNA was synthesized using PrimeScript RT Reagent Kit with gDNA Eraser (TakaRa Beijing, China) in 20 μl reaction mixtures containing random hexamers and oligo dT primers at 37°C for 15 min.

Quantitative real-time PCR (qPCR) was carried out using a CFX Touch Real-Time PCR system (Bio-Rad, California, United States) in 96-well plates with the SYBR Premix EX Taq Kit (TaKaRa, Beijing, China). Each qPCR reaction contained SYBR master mix (5 μl), cDNA template (1 μl), primers (0.4 μl/per primer at 10 μmol), and ddH_2_O (3.2 μl). Reaction conditions were as follows: 95°C for 30 s, 35 cycles at 95°C for 5 s, 58°C for 15 s, and 72°C for 30 s. Reactions were normalized using β-actin, and relative mRNA expression levels were obtained using the 2^–ΔΔct^ method (Livak and Schmittgen, 2001). The primers used for qPCR are shown in Supplementary Table S2.

### Expression in Different Developmental Stages

*Fz2* expression in *C. lividipennis* was estimated by qPCR as described above using primers QFz2-F and QFz2-R (Supplementary Table S2). Total RNA was isolated from eggs (*n* = 50, *N* = 3), first to fifth instar larvae (*n* = 10, *N* = 3) and 1- and 2-day-old female adults (*n* = 5, *N* = 3). Reactions were conducted in a final volume of 10 μl containing 1 μl of the cDNA sample (or standard), 0.2 μl (10 pmol/μl) of each primer and 5 μl SYBR premix Ex Taq. β-actin was amplified from *C. lividipennis* with actin-F and actin-R primers (Supplementary Table S2) and used as an internal control. Standard curves were constructed with 10-fold serial dilutions of cDNA from a pool of 30 individuals as described previously (Chen et al., 2010). Relative mRNA expression was calculated as the means of three individual measurements ± SEM.

### Expression in Different Tissues

To evaluate *Fz2* expression in different tissues, total RNA was extracted from the heads (*n* = 20, *N* = 3), fat bodies (*n* = 15, *N* = 3), midguts (*n* = 30, *N* = 3), ovaries (*n* = 15, *N* = 3), cuticula (*n* = 30, *N* = 3), and legs (*n* = 30, *N* = 3). One or two organs/mirid bug were collected, rinsed in phosphate buffer saline (PBS), and combined with organs from 15–30 individuals as described (Chen et al., 2010). PCR was conducted in a final volume of 20 μl using the reagents, primers, and conditions described above. PCR products (5 μl) were separated by electrophoresis, and gels were stained with ethidium bromide as described previously (Chen et al., 2010).

### Artificial Diets of *C. lividipennis*

Artificial diets of *C. lividipennis* were prepared according to Supplementary Table S3. Compounds were dissolved in ddH_2_O, and the pH was adjusted to 6.4 with 4% KOH in a total volume of 100 ml. Diets were prepared with AAs (normal artificial diet, including six AAs), lacking multiple AAs (-6 AAs, -Arg, -Lys, -Ser, -Pro, -Ala, and -Thr) or lacking individual AAs (-1 AA, -Arg, -Lys, -Ser, -Pro, -Ala, or -Thr). Glass cylinders (7.5 cm × 1.2 cm) were used as feeding chambers. A 50 μl aliquot of the artificial diet was contained between two layers of a Parafilm membrane at the open end of the chamber (the diet capsule), and sterile absorbent cotton was placed at the bottom of the glass cylinder. The diet capsule was replaced every other day, and 200 μl ddH_2_O was added daily to maintain humidity (He et al., 2014). Cylinders were covered with a black cotton cloth, and the open end containing the diet capsule was exposed to light. *C. lividipennis* (10 first instar individuals) was allowed to feed on the diet capsule by puncturing the inner Parafilm membrane, and a total of 10 chambers were used to rear nymphs for each treatment. Rearing was conducted in growth chambers maintained at 26 ± 2°C, 80% RH with a 16:8 h light:dark photoperiod. Mortality was recorded every other day.

### Synthesis of DsRNA and Microinjection

For dsRNA synthesis, a 388 bp fragment of *Fz2* was amplified from cDNA using forward and reverse primers Fz2-F and Fz2-R, respectively (Supplementary Table S2). The reaction conditions were as follows: 35 cycles at 95°C for 30 s, 58°C for 30 s, and 72°C for 45 s, with a final extension at 72°C for 10 min. PCR products were sequenced, and only products where forward and reverse sequences aligned well (98%) were used as templates for dsRNA synthesis. The gene-encoding green fluorescent protein (*GFP*) from Expression vector PHT3AG (ACY56286) was used as control dsRNA, and primers GFP-F and GFP-R were used to amplify the 688 bp fragment containing GFP from Expression vector PHAT3AG plasmid. The T7 RiboMax Express RNAi System (Promega, Madison, WI, United States) was used to prepare dsRNAs. Sense and antisense dsRNAs were generated in separate reactions, precipitated and resuspended in nuclease-free water as described previously (Chen et al., 2010). The quantity and purity of dsRNAs were determined by UV spectroscopy and agarose gel electrophoresis.

*C. lividipennis* was microinjected with dsRNA as described by Wang et al. (2018). In a preliminary study, we evaluated five different dsRNA concentrations, which were designated high (80 and 100 ng), medium (40 and 60 ng), and low (20 ng); 60 ng dsFz2 was selected as an optimum concentration based on the survival rate and RNAi efficiency of *C. lividipennis* (data not shown). Purified dsFz2 (60 ng) was injected into the mesothorax of fifth instar *C. lividipennis* nymphs using a FemtoJet Microinjector (Eppendorf-Nethler-Hinz, Hamburg, Germany); nymphs were then transferred into glass cylinders (7.5 × 1.2 cm) that served as feeding chambers. Diet capsules were replenished every other day. Newly emerged, dsFz2-treated virgin females were then paired with an untreated male. Adult females (*n* = 100) were collected 2 days after emergence (DAE) to determine soluble protein content of ovaries and fat bodies (*n* = 15, *N* = 3 replicates), juvenile hormone (JH) III and ecdysteroid titers (entire body of mated female, *n* = 5, *N* = 3), and body weight (*n* = 10, *N* = 3). Furthermore, the expression of Wnt/β-catenin signaling genes (*Wnt*, *Dvl*, *GSK3*β, β*-catenin*, and *TCF*) was examined, and the number of developed oocytes at 4 DAE (*n* = 10) was assessed. Fat bodies were examined to determine the accumulation of *Vg* mRNA and protein at 2 DAE. Fifty dsFz2-treated, mated adult females were collected at 1, 2, 3, 5, and 7 DAE to examine the efficiency of silencing by RNAi (*n* = 5, *N* = 3). Fifty dsFz2-treated, mated adult females were also dissected to obtain reproductive tracts at 4 DAE (Ge et al., 2020). The longevity of adult females, preoviposition and oviposition periods, and number of eggs laid per mating pair were determined (one dsFz2-treated female mated with an untreated male; and one dsGFP-treated female mated with an untreated male; *n* = 10). The number of eggs laid by the following pairs was also determined: one dsFz2-treated female mated with an untreated control male, one dsTOR-treated (50 ng) female mated with an untreated male, one dsTOR/dsFz2-treated female mated with an untreated male, and one dsGFP-treated female mated with an untreated male (*n* = 10).

### Protein Extraction and Determination

Soluble proteins were extracted from fat bodies and ovaries of 50 dsFz2- and 50 dsGFP-treated mated females using a method similar to that described previously (Ge et al., 2010). Briefly, individual tissues were dissected from adult females at 2 DAE, transferred to microcentrifuge tubes (1.5 ml) and weighed. A 600 μl solution of a NaCl solution [0.4 M NaCl, 1 M phenylmethylsulfonyl fluoride (PMSF)] was added, and tissues were homogenized and centrifuged as described (Ge et al., 2010). The upper lipid layer was filtered with glass fibers, ddH_2_O was added, stored at 4°C overnight, and centrifuged at 4,000 × g at 4°C for 10 min (Ge et al., 2010). Supernatants were removed, and protein sediments were dissolved in 1.0 ml of cold 0.4 M NaCl.

Protein concentrations were determined using the Bradford, 1976 method (1976) and Coomassie Brilliant Blue R250 (Shanghai Chemical Agent Co., Ltd., Shanghai, China). *A*_595_ values were measured by UV spectroscopy, and protein concentrations were determined with a standard curve of bovine serum albumin (BSA) (Shanghai Biochemistry Research Institute, Shanghai, China). Treatments and controls consisted of three independent biological replicates.

### Measurement of JH III and Ecdysteroid Titers

JH III and ecdysteroid titers in mated adult females at 2 DAE were measured by high-performance liquid chromatography (HPLC)/MS (Cornette et al., 2008; Giebbultowicz et al., 2008). JH III and 20-hydroxyecdysone standards were obtained from Sigma-Aldrich (Sigma, United States). Treatments and controls were replicated three times.

### Female Body Mass and Isolation of Ovaries

The weight of 10 dsFz2- and 10 dsGFP-treated mated females were recorded at 2 DAE (Mettler-Toledo EC 100; 0.0001 g sensitivity). Treatments and controls consisted of three independent biological replicates (*n* = 10, *N* = 3). Ovaries (*n* ≥ 10 mated females from dsFz2 and dsGFP treatments at 2 DAE, *N* = 3) were isolated in 0.01 M PBS, fixed in 3.8% formaldehyde, and washed with 0.2% Triton X-100 as described previously (Ge et al., 2019). Images were captured with a Leica DMR connected to a Fuji Fine PixS2 Pro digital camera (Germany).

### Methoprene Treatment

A stock solution (100 μg/μl) of the JH analog (JHA) methoprene (Sigma) was prepared in acetone as described (Ge et al., 2020) and diluted to 10 μg/μl in acetone and ddH_2_O (1:9 v/v). Newly emerged females that had been microinjected with dsFz2 as fifth instar nymphs were selected, and a 1 μl aliquot of the diluted solution was applied topically as described previously (Ge et al., 2020). Treated *C. lividipennis* females were transferred into glass cylinders and maintained on normal artificial diets. Mated, dsFz2-treated *C. lividipennis* females were collected at 2 DAE following the topical application of methoprene, and the expression levels of *Rheb*, *TOR*, *S6K*, and *Vg* were determined by qPCR. Phosphorylation of S6K and synthesis of Vg protein were determined by Western blot (WB) analysis. Treatments and controls consisted of three independent biological replicates.

### Western Blot Analysis

Isolated fat bodies of dsFz2-treated *C. lividipennis* were homogenized in 0.5 ml ice-cold lysis buffer that was supplemented with protease and phosphatase inhibitors as described (Lu et al., 2016). After incubation for 1 h at 4°C, lysates were centrifuged and 30 μg total protein was separated by 10% sodium dodecyl sulfate (SDS)–polyacrylamide gel electrophoresis (PAGE) and transferred to polyvinylidene fluoride (PVDF) membranes; these were treated with blocking buffer and then incubated with primary antibodies as described (Lu et al., 2016). Anti-phospho-p70 S6 kinase (p70-Thr-389 S6K) polyclonal antisera were obtained from Cell Signaling Technology (Danvers, MA, United States), and anti-Vg antisera were prepared by Nanjing Kingsley Biotechnology Co., Ltd. (Nanjing, China). Antisera to β-actin were used as a loading control. After membranes were washed with TBST (Tris-buffered saline, pH 7.4, 0.5% Tween-20), they were incubated in blocking buffer with goat anti-rabbit immunoglobulin G (IgG)-conjugated to horseradish peroxidase secondary antibodies (Sigma) for 1 h at room temperature. Bands were visualized with chemiluminescent substrates and photographed with the GBOX-Chemi XT4 (Syngene, Cambridge, United Kingdom) as described previously (Lu et al., 2016).

### Immunohistochemical Staining

Ovaries from *C. lividipennis* dsFz2- or dsGFP- treated fifth instar nymphs were removed at 4 DAE and fixed in PBS containing 20% surcose and 4% paraformaldehyde (Zhang et al., 2015). Ovaries were washed three times in PBS containing 0.1% Triton X-100 (PBST), blocked in PBST containing 10% fetal bovine serum, and incubated with anti-Vg (1:500) as described previously (Ge et al., 2020). Samples were rinsed three times in PBS (5 min/wash), and a 1:500 dilution of Alexa Fluor 488-labeled goat anti-rabbit antisera (Abbkine, Redlands, CA, United States) was added (Zhang et al., 2015). After a 1 h incubation at ambient temperature, nuclei were counterstained with 100 nM 4′,6-diamidino-2-phenylindole (DAPI) (Beyotime, Shanghai, China) in PBST for 10 min at room temperature. Samples were mounted on glass slides and washed in PBS (3×, 5 min). Fluorescence images were captured with a Zeiss LSM 780 confocal microscope (Carl Zeiss MicroImaging, Göttingen, Germany).

### Predation Capacity of *C. lividipennis*

The predation capacity of *C. lividipennis* was measured as described by Base et al. (2002) with minor modifications. First instar nymphs of BPH (*n* = 20) were placed in glass tubes (3 cm × 25 cm) containing four 15-day-old rice seedlings of rice cv. Ningjin 4, which is susceptible to BPH. Newly emerged (<24 h) *C. lividipennis* adult females were individually placed into glass tubes, which were then sealed with nylon mesh. Surviving BPH nymphs were recorded daily, and the predation number per *C. lividipennis* was accessed based on survival counts of BPH nymphs at days 1 and 3. Treatments and controls consisted of five independent biological replicates.

### Population Growth

Two groups were established to monitor population growth: dsGFP-treated females mated with untreated males (control group), and dsFz2-treated females mated with untreated males (treatment group). A randomized complete block containing five replicates was used as an experimental design. Newly emerged *C. lividipennis* (two pairs) were released on rice plants containing *N. lugens* eggs at the tillering stage (40 days) and enclosed in nylon cylindrical cages as described (Ge et al., 2017). When third instar nymphs of the next generation emerged (approximately 25 days), groups were inspected daily and third instar nymphs were counted; these were transferred to new plastic pots with growing rice plants. Nymphs were examined every 2 days until adults emerged; numbers of both sexes were recorded until the females died. Numbers of adults from the new generation and unhatched egg counts were used to calculate hatch rates and the ratio of adults/adults + unhatched eggs (Ge et al., 2017). The population growth index (PGI) was calculated as described (Ge et al., 2017).

### Statistical Analysis

Normal distributions and homogeneity of variances were confirmed prior to ANOVA as described (Ge et al., 2017). A one-way ANOVA was conducted for soluble proteins (ovaries and fat bodies), body weight, JH III and ecdysone titers, longevity, preoviposition and oviposition periods, number of developed oocytes, numbers of eggs laid and offspring, hatching rate, gender ratio, and mRNA expression levels. Unless otherwise noted, data are expressed as means ± standard error (SEM) from three independent biological replicates. Two-way (days after emergence and dsRNA treatment) ANOVAs were used to analyze the data. Multiple comparisons of the means were calculated with Fisher’s protected least significant difference test. All analyses were conducted using the data processing system of Tang and Feng (2007).

## Results

### Amino Acid Concentrations in Rice and Gramineous Species

The free AA content of five gramineous species (*D. ciliaris*, *C. dactylon*, *L. chinensis*, *E. indica*, and *E. glabrescens*) and rice were analyzed and compared (Supplementary Table S1). The concentrations of Ala, Lys, Pro, Arg, Ser, and Thr were significantly higher in rice than in the five gramineous species; an exception was the higher Lys content in *E. glabrescens* as compared to rice (Supplementary Table S1).

### Frizzled 2 Expression Levels in *C. lividipennis*

**FIGURE 1:**
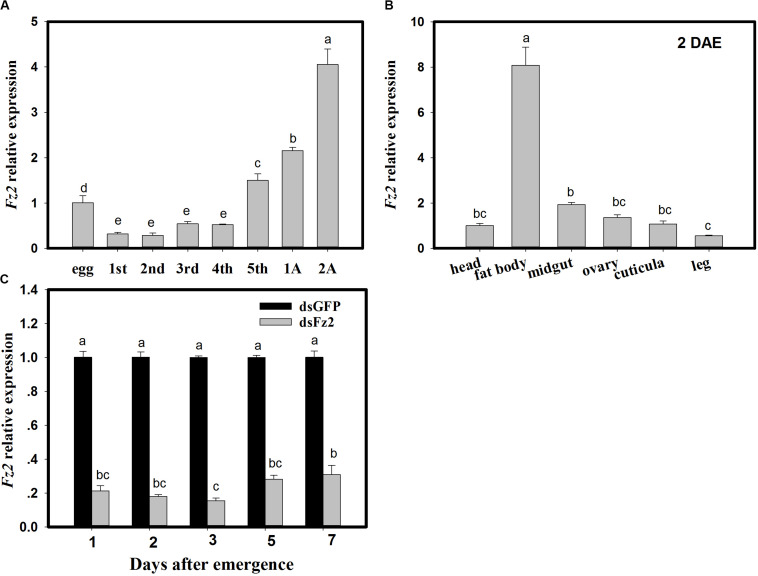
*Frizzled 2* (*Fz2*) expression profiles and RNAi silencing efficiency in *Cyrtorhinus lividipennis* females. Panels show the mean expression of *Fz2* at different development stages **(A)** and in different tissues **(B)**. **(C)** Shows *Fz2* expression in dsFz2- and dsGFP-treated *C. lividipennis* females at different days after emergence (DAE), which is an indicator of RNAi-mediated silencing efficiency. The data are from three independent biological replicates and are presented as mean ± SEM.

With respect to developmental stages, *Fz2* expression increased in fifth instar nymphs and adult *C. lividipennis* (*F* = 213.8; df = 7, 23; *P* = 0.0001) and was 49∼132% higher than that in egg (Figure 1A). *Fz2* was highly expressed (7-fold higher) in the fat body of *C. lividipennis* female adults at 2 DAE (*F* = 212.2; df = 5, 17; *P* = 0.0001) (Figure 1B). In *C. lividipennis* females treated with 60 ng dsFz2, expression of *Fz2* was 69∼84% lower than that in dsGFP-treated females at 1, 2, 3, 5, and 7 DAE (Figure 1C).

### Amino Acid Signals Mediate Transcriptional Changes in the *Wnt/*β*-Catenin* Pathway

**FIGURE 2:**
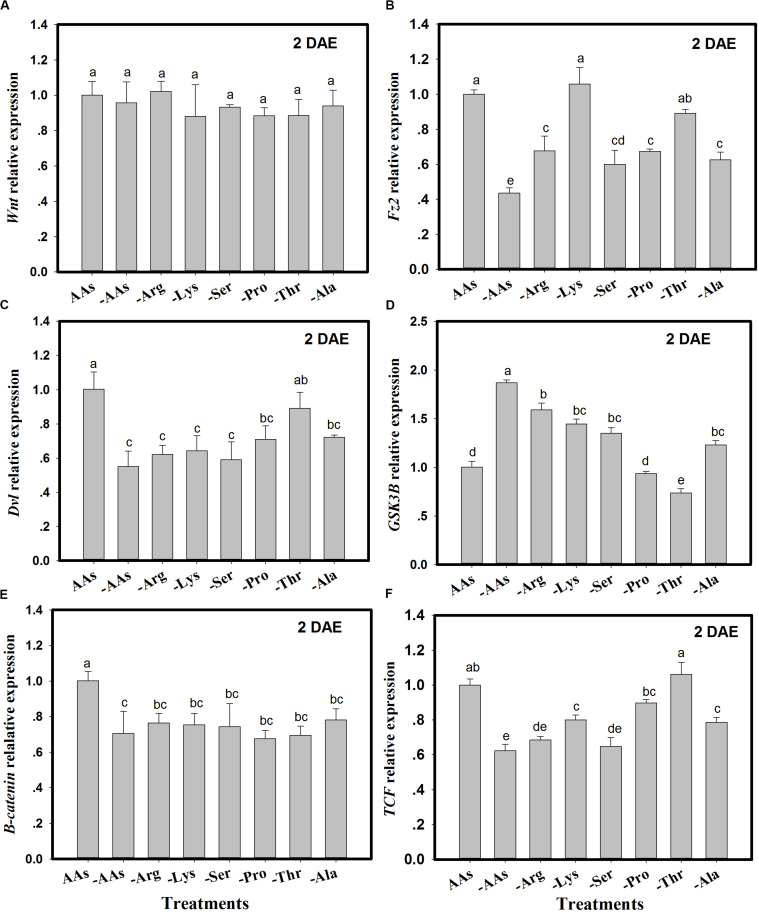
The expression of *Wnt* pathway genes in *Cyrtorhinus lividipennis* females fed on artificial diets lacking one or more amino acids (AAs). **(A–F)** Show the expression of *Wnt*, *Fz2*, *Dvl*, *GSK3*β, β*-catenin*, and *TCF* in females fed on artificial diets containing all essential AAs or lacking one or of the following AAs: Arg, Lys, Ser, Pro, Thr, and Ala. Relative mRNA levels were determined by comparison with standard curves using each gene and normalized using β*-actin*. Data points are from three independent biological replicates and are shown as mean ± SEM.

The expression of Wnt pathway genes (*Wnt*, *Fz2*, *Dvl*, *GSK3*β, β*-catenin*, and *TCF*) was monitored in *C. lividipennis* females fed on artificial diets lacking one or more AAs (Figure 2). There were no significant differences in *Wnt* expression in *C. lividipennis* fed on various artificial diets (*F* = 0.9; df = 7, 23; *P* = 0.4901) (Figure 2A). However, *Fz2* was differentially expressed in *C. lividipennis* fed on diets lacking one or more AAs (Figure 2B). A significant reduction in expression was observed for insects fed on diets lacking all six AAs (↓56%) or lacking Arg (↓32%), Ser, (↓40%), Pro (↓33%), or Ala (↓37%) (*F* = 24.7; df = 7, 23; *P* = 0.0001). No significant differences were noted for *Fz2* expression on diets lacking Lys or Thr. The expression of *Dvl* showed a 45% decrease when insects were fed on diets lacking all six AAs; reductions were ↓38% for diets lacking Arg (-Arg), ↓36% for -Lys, ↓41% for -Ser, ↓29% for Pro, and ↓28% for Ala (*F* = 10.7; df = 7, 23; *P* = 0.0001). *Dvl* expression level was not significantly altered in diets lacking Thr (Figure 2C). Expression levels of *GSK3*β were upregulated in diets lacking the six AAs and in diets lacking Arg, Lys, Ser, and Ala. *GSK3*β expression was downregulated in diets lacking Thr and was not significantly altered in diets lacking Pro (Figure 2D). β*-catenin* showed significant decreases in expression (Figure 2E) when one or more AAs were absent; e.g., ↓29% for diets lacking all six AAs, ↓23% for diets lacking Arg (Arg-), ↓25% for Lys-, ↓26% for Ser-, ↓32% for Pro-, ↓31% for Thr-, and ↓22% for Ala- (*F* = 7.7; df = 7, 23; *P* = 0.0004). *TCF* expression was reduced in insects fed on diets lacking the six AAs (↓27%) and in diets lacking Arg (↓32%), Lys (↓20%), Ser (↓35%), and Ala (↓21%) (*F* = 36.5; df = 7, 23; *P* = 0.0001) (Figure 2F).

### Silencing Frizzled 2 Inhibits Wnt and Target of Rapamycin Pathways

**FIGURE 3:**
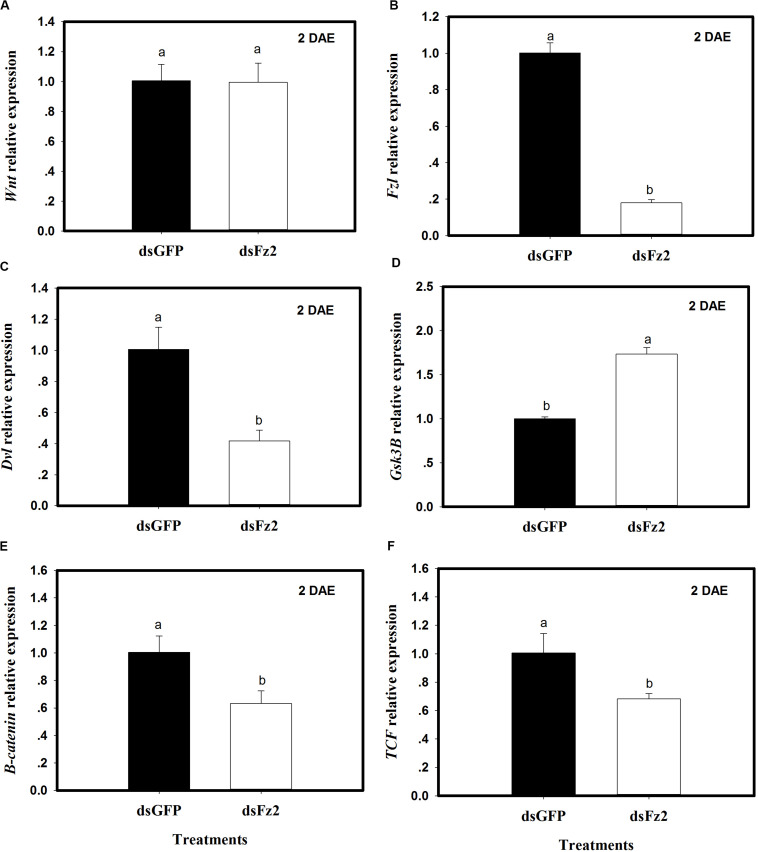
Silencing *Frizzled 2* (*Fz2*) results in reduced expression of selected genes in the *Wnt* signaling pathway. **(A–F)** Show mean expression levels of *Wnt*, *Fz2*, *Dvl*, GSK3β, β-catenin, and TCF, respectively, in dsGFP- and dsFs2-treated *Cyrtorhinus lividipennis* at 2 days after emergence (DAE). Relative mRNA levels were determined as stated above and normalized with β*-actin*. Data points are from three independent biological replicates and represent mean ± SEM.

The expression of selected *Wnt* pathway genes was analyzed in *C. lividipennis* females treated with dsFz2 or dsGFP. No significant differences were observed in *Wnt* transcription levels in dsFz2- and dsGFP-treated insects (Figure 3A). However, *C. lividipennis* treated with dsFz2 showed a significant reduction in the expression of *Fz2* (Figure 3B; ↓82%: *F* = 595.1; df = 1, 5; *P* = 0.0001), *Dvl* (Figure 3C; ↓59%: *F* = 42.0; df = 1, 5; *P* = 0.0029), β*-catenin* (Figure 3E; ↓37%: *F* = 18.7; df = 1, 5; *P* = 0.0001), and *TCF* (Figure 3F; ↓32%: *F* = 15.6; df = 1, 5; *P* = 0.0167) relative to dsGFP treatments at 2 DAE. Interestingly, dsFz2-treated *C. lividipennis* showed significant upregulated levels of *GSK3*β (Figure 3D;↑73%: *F* = 274.1; df = 1, 5; *P* = 0.0001) relative to dsGFP-treated controls.

**FIGURE 4:**
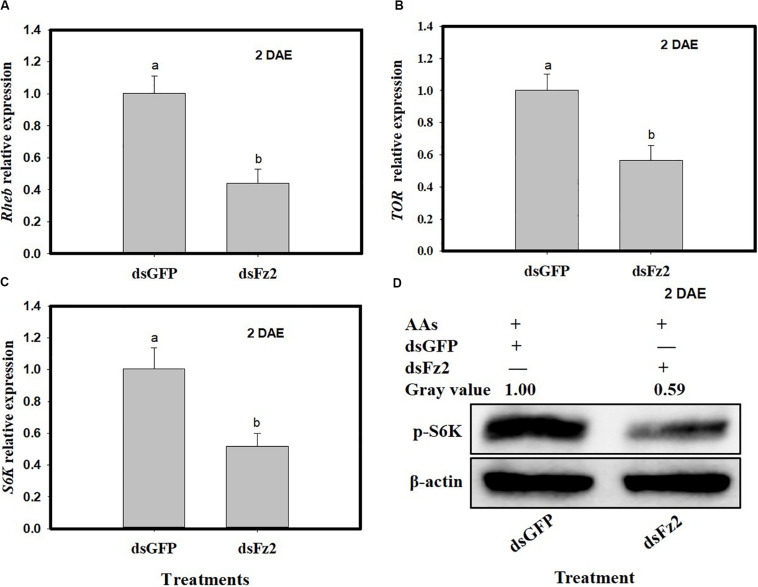
Silencing *Frizzled 2* (*Fz2*) inhibits the expression and activity of selected components in the target of rapamycin (TOR) signaling pathway. **(A–C)** Show the mean expression levels of *Rheb*, *TOR*, and *S6K* at 2 days after emergence (DAE), respectively, in dsGFP- and dsFs2-treated *Cyrtorhinus lividipennis*. Relative mRNA levels were determined by comparison with standard curves using each gene and normalized using β*-actin*. **(D)** Shows the phosphorylation of S6K in fat body isolated from dsGFP- and dsFz2-treated *C. lividipennis* at 2 DAE. Phosphorylation was detected by Western blot analysis using *phospho*-p70-Thr-389 S6K polyclonal antisera; the loading control (β-actin) was analyzed using anti-β-actin antisera. Expression data points are from three independent biological replicates and are presented as the mean ± SEM.

*C. lividipennis* females treated with dsFz2 also showed reduced transcription of *Rheb* (*F* = 49.4; df = 1, 5; *P* = 0.0001), *TOR* (*F* = 30.9; df = 1, 5; *P* = 0.0051), and *S6k* (*F* = 30.3; df = 1, 5; *P* = 0.0001), which were 56, 44, and 49% lower than dsGFP-treated *C. lividipennis* at 2 DAE, respectively (Figures 4A–C). Western blot analysis confirmed that the fat bodies in dsFz2-treated *C. lividipennis* females had lower levels (∼41%) of S6K phosphorylation than dsGFP-treated insects (Figure 4D). Thus, *Fz2* silencing results in significant inhibition of selected genes in the Wnt/β-catenin and TOR pathways.

### *Frizzled 2* Silencing Inhibits Vg Expression

**FIGURE 5:**
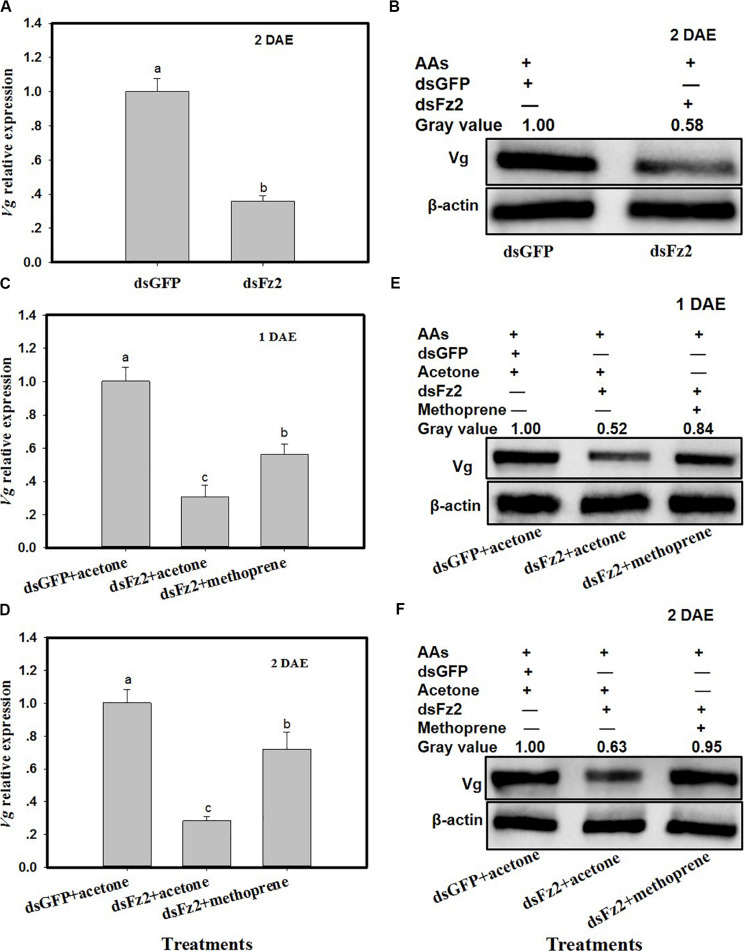
Frizzled 2 (Fz2) silencing inhibits the expression and production of Vg in the *Cyrtorhinus lividipennis* fat body. **(A,B)** Show the mean *Vg* expression and Vg protein levels at 2 days after emergence (DAE) in dsGFP- and dsFz2-treated *C. lividipennis* as determined by quantitative real-time PCR (qPCR) and Western blot analysis, respectively. **(C,D)** Show *Vg* expression at 1 DAE and 2 DAE. **(E,F)** Show Vg protein production as measured by Western blot analysis with antibody against β-actin as a loading control at 1 DAE and 2 DAE after methorprene and acetone treatments with new emergence dsGFP- and dsFz2-treated female adult. The relative mRNA levels were determined by comparison with the standard curve of the gene concerned and normalized against the β*-actin* gene. β-actin antibody was used as the loading control. The relative gray values normalized to β-actin were marked above corresponding bands. Expression data are from three independent biological replicates and are presented as the mean ± SEM.

*C. lividipennis* females treated with dsFz2 exhibited ∼64% lower *Vg* expression as compared to dsGFP-treated females at 2 DAE (Figure 5A; *F* = 184.7; df = 1, 5; *P* = 0.0001). As expected, dsFz2-mediated silencing also depleted the amount of Vg in the fat body of *C. lividipennis* female adults, which was approximately 42% lower than that in dsGFP-treated females at 2 DAE (Figure 5B). Supplementation of dsFz2-treated females with the JH analog methoprene partially rescued *Vg* expression at 1 DAE and 2 DAE (*F* = 68.6; df = 1, 5; *P* = 0.0001 for 1 DAE and *F* = 68.8; df = 1, 5; *P* = 0.0001 for 2 DAE) (Figures 5C,D), with levels 0.82- and 1.54-fold, respectively, of the amount of Vg in the dsFz2 + acetone treatment. The application of methoprene to the dsFz2 treatment partially restored Vg protein abudance with 0.60- and 0.51-fold at 1 DAE and 2 DAE, respectively, of the levels of Vg present in the dsFs2 + acetone treatment (Figures 5E,F).

### *Frizzled 2* Silencing Has Multiple Effects on *C. lividipennis* Adult Females

**FIGURE 6:**
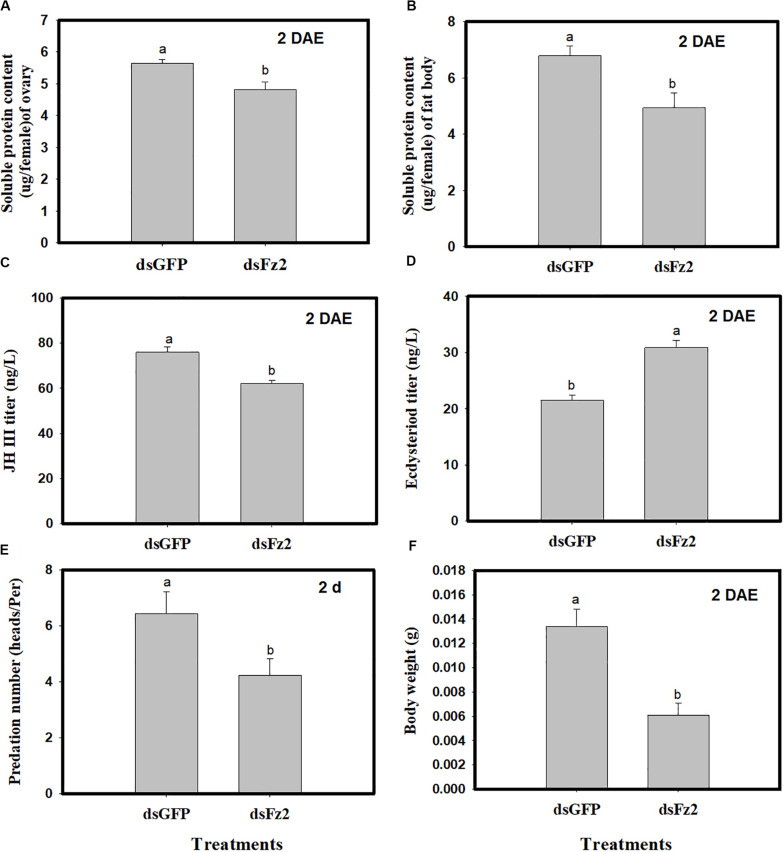
Effect of *Frizzled 2* (*Fz2*) silencing on soluble proteins, JH III and ecdysteroid titers and predation. *Cyrtorhinus lividipennis* female adults were treated with dsGFP (control) or dsFz2, and data were analyzed 2 days after emergence (DAE). **(A,B)** Show the mean soluble protein in ovaries and fat bodies, respectively. **(C,D)** Represent mean JH III and ecdysteroid titers; and **(E,F)** indicate mean predation number and body weight, respectively. The data shown are from three independent biological replicates and are presented as the mean ± SEM.

Dietary dsFz2 treatment led to significant decreases in soluble protein content of ovaries and fat bodies, which were 15% (*F* = 9.4; df = 1, 5; *P* = 0.0372) and 27% lower (*F* = 25.3; df = 1, 5; *P* = 0.0073) than dsGFP treatment at 2 DAE, respectively (Figures 6A,B). RNAi-mediated depletion of *Fz2* decreased JH III titers (↓18%: *F* = 22.1; df = 1, 5; *P* = 0.0093) and increased ecdysteroid titers (↑43%: F = 113.5; df = 1, 5; *P* = 0.0004) at 2 DAE (Figures 6C,D). The knockdown of *Fz2* led to decreased predation capacity for *C. lividipennis* female adults (↓34%: *F* = 25.6; df = 1, 5; *P* = 0.0003) compared to dsGFP-treated females at 2 DAE (Figure 6E); furthermore, *Fz2* silencing caused a significant decline (↓54%: *F* = 18.7; df = 1, 5; *P* = 0.0124) in body weight compared to dsGFP at 2 DAE (Figure 6F).

### *Frizzled 2* Silencing Impacts Reproduction of *C. lividipennis* Females

**FIGURE 7:**
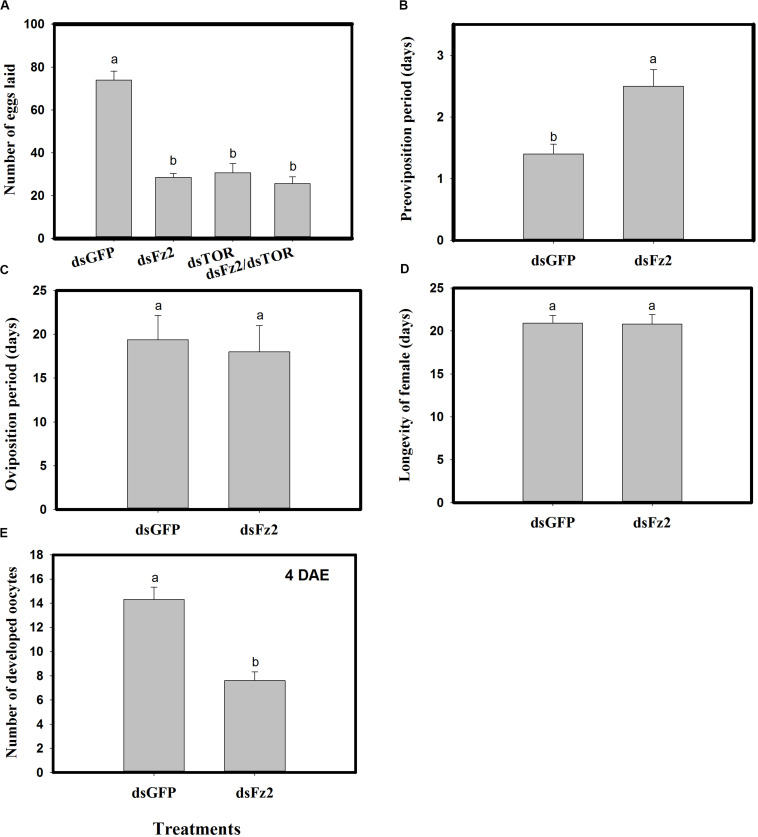
RNAi-mediated silencing of *Frizzled 2* (*Fz2*) influences reproductive parameters in *Cyrtorhinus lividipennis*. **(A)** Shows the mean number of eggs laid by fifth instar nymphs injected with dsRNA of *GFP* (dsGFP), *TOR* (dsTOR), *Fz2* (dsFz2), or dsFz2/dsTOR. **(B–E)** Show mean preoviposition period, oviposition period, longevity, and number of developed oocytes, respectively. The data paints are from three independent biological replicates and are presented as mean ± SEM.

Dietary dsFz2, dsTOR, and dsFz2/dsTOR (Figure 7A) resulted in significantly lower numbers of eggs laid as compared to the dsGFP treatment group (dsFz2 ↓61%; dsTOR ↓58%; dsFz2/dsTOR ↓65%; *F* = 95.7; df = 3, 39; *P* = 0.0001). RNAi-mediated depletion of *Fz2* resulted in a prolonged preoviposition period (↑78%; *F* = 12.2; df = 1, 19; *P* = 0.0026) (Figure 7B). However, there were no significant differences in dsFz2- and dsGFP-treated females for the oviposition period (*F* = 1.2; df = 1, 19; *P* = 0.2837) or longevity (*F* = 0.001; df = 1, 19; *P* = 0.9454) (Figures 7C,D). The depletion of *Fz2* resulted in a significant reduction in oocyte development (↓47%, *F* = 28.3; df = 1, 19; *P* = 0.0001) as compared to dsGFP treatment (Figure 7E). Since the number of eggs produced by dsFz2/dsTOR-treated *C. lividipennis* showed no significant difference from that of dsFz2- or dsTOR-treated females (Figure 7A), the data suggest that *Fz2* and *TOR* regulate egg production *via* a shared pathway.

### *Frizzled 2* Silencing Inhibits Oocyte Development and Vg Uptake

**FIGURE 8:**
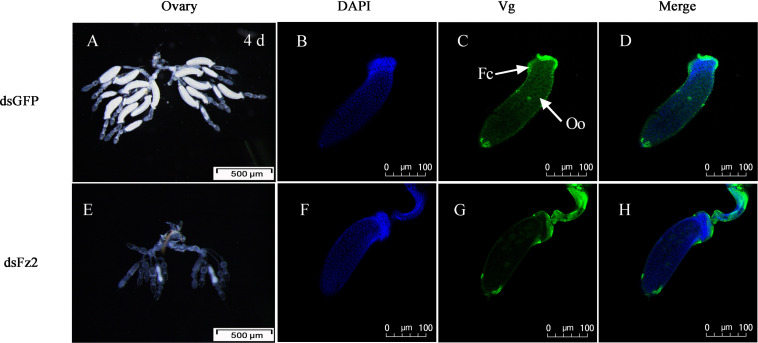
Effects of dsFz2 treatment on ovary development in dsGFP- and dsFz2-treated *Cyrtorhinus lividipennis* females at 4 days after emergence (DAE). **(A)** Ovaries of a female (fourth day after emerence), new-emergence female adult were treated with dsFz2 (approximately 60 ng per insect; *n* ≥ 10, *N* = 3); **(E)** ovaries of a control female adult treated with dsGFP at the newly emerged. Ovaries were observed under a stereomicroscope. Scale bar, 500 μm. The fluorescence images were acquired using a Zeiss LSM 780 confocal microscope. Ovaries were incubated with anti-vitellogenin (Vg, 1:500) conjugated to Alex Fluor 488-labeled goat anti-rabbited immunoglobulin G (IgG). **(B,F)** Show nuclear staining with 4’,6-diamidino-2-phenylindole (DAPI) (blue). **(C,G)** Show Vg (green) staining. Two stains were merged to localize Vg (**D,H**). Fc, follicular cell; Oo, oocyte. Scale bar, 100 μm.

There were no obvious external morphological changes in *C. lividipennis* females after dsFz2 treatment (data not shown). In the ovaries of the dsGFP-treated control group, every ovariole contained one or two fully developed banana-shaped oocytes at 4 DAE (Figure 8A). However, dsFz2 treatment resulted in undeveloped ovaries and limited oocyte growth (Figure 8E). No fully developed oocytes were observed in dsFz2-treated females at 5 DAE (data not shown). To obtain more details on the phenotype of *Fz2* silenced females, immunohistochemical (IHC) staining was performed. The results of IHC staining showed that the uptake of vitellin by the oocytes was inhibited when Fz2 was depleted (Figure 8G) as compared to the dsGFP treatment (Figure 8C). We scarcely observed scattered Vg and no patency was spotted in the dsFz2 treatment (Figure 8G).

### *Frizzled 2* Silencing Inhibits Embryonic Development

**FIGURE 9:**
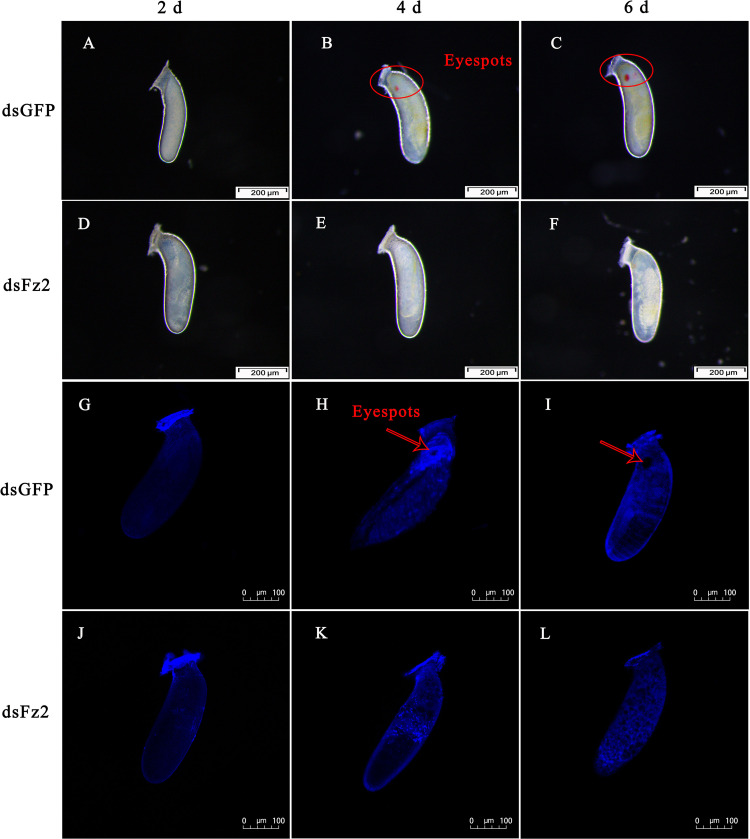
Effects of dsFz2 treatment on eggs and embryonic development at 2, 4, and 6 days after emergence (DAE). **(A–C)** Show the phenotype of eggs produced by mated *Cyrtorhinus lividipennis* females treated with dsGFP at 2, 4, and 6 DAE, respectively. **(D–F)** Show eggs laid by mated C. *lividipennis* injected with dsFz2 at 2, 4, and 6 DAE, respectively. In **(G–I)**, the nuclear stain 4’,6-diamidino-2-phenylindole (DAPI) was used to stain eggs in the dsGFP-treated females at 2, 4, and 6 DAE, respectively. **(J–L)** Show eggs of dsFz2-treated females stained with DAPI at 2, 4, and 6 DAE, respectively. Fluorescence images were acquired using a Zeiss LSM 780 confocal microscope. Red ovals and arrows indicate the location of eyespots. Scale bar, 100 or 200 μm.

The phenotype of eggs laid by dsFz2-treated females of *C. lividipennis* was examined at 2, 4, and 6 DAE (Figure 9). Eggs laid by dsGFP- and dsFz2-treated females showed no obvious morphological differences at 2 DAE (Figures 9A,D). Eyespots were observed at days 4 and 6 in eggs of dsGFP-treated females (Figures 9B,C); however, eyespots were absent in the dsFz2-treated embryos (Figures 9E,F). DAPI staining confirmed the eyespots in eggs laid by dsGFP-treated females at 4 and 6 DAE (Figures 9H,I) and the absence of eyespots in dsFz2-treated embryos (Figures 9K,L).

### Effects of *Frizzled 2* Silencing on Numbers of Offspring, Hatching Rate, Gender Ratio, and Population Growth Index

Dietary supplementation with dsFz2 led to reduced numbers of offspring (*F* = 27.5; df = 1, 9; *P* = 0.0001), hatching rates (*F* = 38.7; df = 1, 9; *P* = 0.0003), and PGI values (*F* = 29.8; df = 1, 9; *P* = 0.0001), which were 53, 30, and 34% lower, respectively, than those in dsGFP-treated females (Table 1). However, no significant difference in gender ratios was detected between dsGFP- and dsFz2-treated females (*F* = 0.1; df = 1, 9; *P* = 0.7651).

**TABLE 1:** Effects of *Frizzled 2* (*Fz2*) silencing on the number of offspring, hatching rate, gender ratio, and population growth index (PGI).

**Treatments**	**Number of offspring**	**Hatching rate**	**Gender ratio**	**PGI**
dsGFP♀ × control♂	230 ± 46.1a	0.78 ± 0.05a	1.14 ± 0.11a	36.7 ± 5.6a
dsFz2♀ × control♂	106.2 ± 25.9b	0.55 ± 0.07b	1.09 ± 0.09a	24.3 ± 3.2b

*Means ± SE of five replicates. Means within columnns followed by different letters are followed by different letters are significantly different at the 5% level (*P* < 0.05).

## Discussion

This study demonstrates the critical role of *Fz2* in the fertility of the predator *C. lividipennis*. *Fz2* activated the Wnt/β-catenin and TOR signaling pathways and modulated embryonic development, oogenesis, and reproduction. *Fz2* expression steadily increased from the fifth nymph stage to adults at 2 DAE and was highly expressed in fat bodies. When *C. lividipennis* was fed on AA-deprived artificial diets, expression of Wnt pathway components was altered, although *Wnt* expression was not directly impacted (Figure 2). Furthermore, RNAi-mediated depletion of *Fz2* inhibited the expression of selected genes in the *Wnt* and *TOR* signaling pathways (Figures 3, 4).

The conserved TOR pathway detects nutritional signals and mediates S6K phosphorylation, which results in vitellogenesis (Attardo et al., 2003; Park et al., 2006). In *C. lividipennis*, RNAi-mediated depletion of *Fz2* decreased *Vg* expression, Vg protein synthesis, and S6K phosphorylation, indicating that *Fz2* was a key component in the TOR signaling pathway. Vitellogenesis is the process of accumulating Vg and other compounds into developing oocytes (Davey et al., 1994; Sappington and Raikhel, 1998). When oocytes are fully developed, they are coated with chorion and become “eggs” in ovarioles (Ramaswamy et al., 1990). We show that *Fz2* silencing inhibited TOR signal transduction and S6K phosphorylation, resulting in reduced Vg protein synthesis and impaired oocyte development.

In insects, a variety of endocrine signals are associated with the development of ovaries (Roy et al., 2018); one example is JH, which prevents premature metamorphosis (Jindra et al., 2013). In the current study, the addition of the JH analog methoprene to dsFz2 treatments rescued both *Vg* expression and Vg protein synthesis (Figure 5), thus supporting the role of *Fz2* in the regulation of *C. lividipennis* vitellogenesis. Furthermore, silencing *Fz2* decreased soluble proteins in ovaries and fat bodyies, JH III titers, and numbers of developed oocytes and eggs laid (Figures 6, 7). In various adult insects, JH stimulates the development of ovaries *via* gonadotropin (Riddiford, 1994; Wu et al., 2018; Guo et al., 2018), enhances Vg biosythesis (Satyanarayana et al., 1994; Wyatt and Davey, 1996), and functions to modulate the endocrine system (Oberlander et al., 1995; Martin et al., 2001). JH usually facilitates vitellogenesis in oocytes by inducing follicular patency (Sevala and Davey, 1989; Kim et al., 1999; Jing et al., 2018); furthermore, insect fertility is strongly correlated with the number of ovarioles harboring oocytes (Roitberg et al., 2001). In this study, RNAi-mediated depletion of *Fz2* impacted fecundity by inhibiting ovarioles, oocytes, and embryonic development (Figures 7–9).

Egg production by dsFz2/dsTOR-treated *C. lividipennis* was not significantly different from egg numbers produced by dsFz2- or dsTOR-treated *C. lividipennis*, which suggests that Fz2 and TOR regulate egg production *via* a shared pathway. In several insect species, the regulation of JH biosynthesis by nutritional signals and the TOR pathway has been established (Tatar et al., 2001; Tu et al., 2005). For example, The TOR pathway in *Blattella germanica* was proposed to function in Vg production along with nutritional signals and JH biosynthesis (Maestro et al., 2009). In vertebrate organisms, Wnt signaling cooperated with mutliple hormone signaling pathways to modulate development (Wang et al., 2007); for example, the Wnt pathway interacted with estrogen to control transcription in osteoblasts (Kouzmenko et al., 2004; Lieder et al., 2010). In insects, both Wnt and JH signaling control many developmental processes (Abdou et al., 2011). In this study, the depletion of *Fz2* by RNAi led to reduced development of ovaries and embryos and reduced Vg uptake in oocytes. In *Drosophila*, *Fz* was required for tissue polarity in epidermal cells (Gubb, 1993). Furthermore, in *A. aegypti*, *AaFz2* responded to AA signals to stimulate fecundity, and the TOR and Wnt signaling pathways interacted synergistically in vitellogenesis (Weng and Shiao, 2015). The Wnt pathway plays a pivotal role in regulating diapause in *Bombyx mori* (Lin et al., 2009) and regulates *Helicoverpa armigera* pupal development (Chen and Xu, 2014). Our study establishes a link between Wnt and TOR pathways, which interact synergistically to regulate *C. lividipennis* reproduction in response to AA signals.

In summary, we show that *Fz2* responds to AA signals that are transduced through the Wnt and TOR pathways and participates in embyonic development, oogensis, and egg production of *C. lividipennis* females. Furthermore, we demonstrate a cross talk between the Fz2 and TOR pathways in *C. lividipennis* reproduction. These results provide new insight into large-scale rearing of *C. lividipennis*, and this may ultimately improve the ability to deploy this predator for BPH control in rice fields.

## Data Availability Statement

The original contributions presented in the study are included in the article/Supplementary Material, further inquiries can be directed to the corresponding author.

## Author Contributions

LG designed the research. LJ, SZ, YZ, and QW conducted the experiments. SZ and YZ performed the data analysis. LG wrote the first draft of the manuscript. LG and FL revised the final draft of the manuscript. All authors contributed to the article and approved the submitted version.

## Conflict of Interest

The authors declare that the research was conducted in the absence of any commercial or financial relationships that could be construed as a potential conflict of interest.

## Supplementary Material

TABLE S1 Comparison of free amino acids in rice (*Oryza sativa*) and five gramineous species.

TABLE S2 PCR primers used in this study.

TABLE S3 Preparation of holomorphic artificial diet for *C. lividipennis*.
