# Supplementary material for: Frizzled 2 Functions in the Regulation of TOR-Mediated Embryonic Development and Fecundity in Cyrtorhinus lividipennis Reuter
Source: Front Physiol. 2020 Sep 16;11:579233. doi: 10.3389/fphys.2020.579233 (PMC7526694; doi:10.3389/fphys.2020.579233)
Supplement: TABLE S1 — Comparison of free amino acids in rice (Oryza sativa) and five gramineous species. [file Table_1.DOCX]

**Table S1** Comparison of free amino acids in rice (*Oryza sativa*) and five gramineous species.

Table S2 PCR primers used in this study.

**Table S3** Preparation of holomorphic artificial diet for *C. lividipennis*

| **Essential amino acid**  **(2×)** | **g/100 mL** | **Nonessential amino acid**  **(2×)** | **g/100 mL** |
| --- | --- | --- | --- |
| Met | 0.14 | Gly | 0.23 |
| Arg | 0.54 | Ala | 0.35 |
| Lys | 0.3 | Asp | 0.16 |
| Leu | 0.2 | Asn | 0.46 |
| Ile | 0.35 | Cystine | 0.04 |
| Thr | 0.56 | Cys | 0.02 |
| Val | 0.2 | Glu | 0.35 |
| Phe | 0.21 | Gln | 0.48 |
| Trp | 0.3 | Pro | 0.24 |
| His | 0.22 | Ser | 0.66 |
|  |  | Tyr | 0.02 |
| **Vitamins (10×)** | **g/100 mL** |  |  |
| calcium pantothenate | 0.05 | r-aminobutyric acid-GABA | 0.02 |
| riboflavin | 0.005 | **Inorganic salts (100×)** | **g/100 mL** |
| inositol | 0.5 | CaCl_2_.2H_2_O | 0.3115 |
| thiamine | 0.025 | CuCl_2_.2H_2_O | 0.0268 |
| biotin | 0.0005 | MnCl_2_.4H_2_O | 0.0793 |
| nicotinic acid | 0.15 | ZnCl_2_ | 0.0396 |
| pyridoxine hydrochloride | 0.025 | KH_2_PO_4_ | 0.5 |
| choline chloride | 0.5 | MgCl_2_.6H_2_O | 0.2 |
| folic acid | 0.005 | FeCl_3_ | 0.0003 |
| vitamin C | 1 | **sucrose** | 9 |
|  |  | pH | 6.4 |
